# Supplementary material for: Allosteric regulation and crystallographic fragment screening of SARS-CoV-2 NSP15 endoribonuclease
Source: Nucleic Acids Res. 2023 Apr 28;51(10):5255–70. doi: 10.1093/nar/gkad314 (PMC10250223; doi:10.1093/nar/gkad314)
Supplement: gkad314_Supplemental_Files [file gkad314_supplemental_files.zip › Supplementary datasets.zip!/188/Refine_9/01-BUSTER/Cycle-1/shell.01/R_Rfree.html]

# Rxpct factors in resolution bins at cycle 0


|  |  |  |  |  |  |  |  |  |
| --- | --- | --- | --- | --- | --- | --- | --- | --- |
|  | **<d> (Å)**  Rxpct\_Work | Rxpct\_Free | Rxpct\_All | |  |  |  |  |
|  | 16.0655 | 0.3805 | 0.5032 | 0.3845 |  |
|  | 9.2754 | 0.2232 | 0.2612 | 0.2254 |  |
|  | 7.1847 | 0.2057 | 0.2473 | 0.2081 |  |
|  | 6.0722 | 0.2177 | 0.2771 | 0.2203 |  |
|  | 5.3552 | 0.1839 | 0.2811 | 0.1879 |  |
|  | 4.8439 | 0.1722 | 0.2215 | 0.1751 |  |
|  | 4.4558 | 0.1700 | 0.2193 | 0.1726 |  |
|  | 4.1481 | 0.1701 | 0.2230 | 0.1732 |  |
|  | 3.8965 | 0.1795 | 0.2343 | 0.1821 |  |
|  | 3.6857 | 0.1821 | 0.2470 | 0.1850 |  |
|  | 3.5058 | 0.1858 | 0.2268 | 0.1878 |  |
|  | 3.3499 | 0.1898 | 0.2453 | 0.1929 |  |
|  | 3.2131 | 0.2024 | 0.2240 | 0.2037 |  |
|  | 3.0918 | 0.2035 | 0.2489 | 0.2055 |  |
|  | 2.9833 | 0.2040 | 0.2399 | 0.2055 |  |
|  | 2.8855 | 0.2119 | 0.2486 | 0.2138 |  |
|  | 2.7967 | 0.2102 | 0.2582 | 0.2123 |  |
|  | 2.7156 | 0.2181 | 0.2211 | 0.2182 |  |
|  | 2.6412 | 0.2729 | 0.2774 | 0.2732 |  |
|  | 2.5725 | 0.3722 | 0.2553 | 0.3684 |  |
|  | **Overall** | 0.2009 | 0.2481 | 0.2033 |  |

# Rxpct factors in resolution bins at cycle 1


|  |  |  |  |  |  |  |  |  |
| --- | --- | --- | --- | --- | --- | --- | --- | --- |
|  | **<d> (Å)**  Rxpct\_Work | Rxpct\_Free | Rxpct\_All | |  |  |  |  |
|  | 16.0655 | 0.3817 | 0.5021 | 0.3857 |  |
|  | 9.2754 | 0.2222 | 0.2605 | 0.2245 |  |
|  | 7.1847 | 0.2043 | 0.2499 | 0.2069 |  |
|  | 6.0722 | 0.2166 | 0.2719 | 0.2190 |  |
|  | 5.3552 | 0.1808 | 0.2716 | 0.1846 |  |
|  | 4.8439 | 0.1691 | 0.2175 | 0.1720 |  |
|  | 4.4558 | 0.1674 | 0.2199 | 0.1701 |  |
|  | 4.1481 | 0.1679 | 0.2214 | 0.1710 |  |
|  | 3.8965 | 0.1773 | 0.2362 | 0.1801 |  |
|  | 3.6857 | 0.1810 | 0.2463 | 0.1839 |  |
|  | 3.5058 | 0.1841 | 0.2206 | 0.1859 |  |
|  | 3.3499 | 0.1891 | 0.2470 | 0.1924 |  |
|  | 3.2131 | 0.2028 | 0.2247 | 0.2041 |  |
|  | 3.0918 | 0.2024 | 0.2488 | 0.2045 |  |
|  | 2.9833 | 0.2027 | 0.2390 | 0.2043 |  |
|  | 2.8855 | 0.2118 | 0.2449 | 0.2135 |  |
|  | 2.7967 | 0.2103 | 0.2574 | 0.2124 |  |
|  | 2.7156 | 0.2195 | 0.2271 | 0.2199 |  |
|  | 2.6412 | 0.2751 | 0.2813 | 0.2756 |  |
|  | 2.5725 | 0.3714 | 0.2564 | 0.3676 |  |
|  | **Overall** | 0.1995 | 0.2468 | 0.2019 |  |

# Rxpct factors in resolution bins at cycle 2


|  |  |  |  |  |  |  |  |  |
| --- | --- | --- | --- | --- | --- | --- | --- | --- |
|  | **<d> (Å)**  Rxpct\_Work | Rxpct\_Free | Rxpct\_All | |  |  |  |  |
|  | 16.0655 | 0.3866 | 0.5096 | 0.3906 |  |
|  | 9.2754 | 0.2248 | 0.2618 | 0.2270 |  |
|  | 7.1847 | 0.2037 | 0.2458 | 0.2060 |  |
|  | 6.0722 | 0.2150 | 0.2719 | 0.2175 |  |
|  | 5.3552 | 0.1786 | 0.2722 | 0.1824 |  |
|  | 4.8439 | 0.1665 | 0.2162 | 0.1694 |  |
|  | 4.4558 | 0.1656 | 0.2213 | 0.1685 |  |
|  | 4.1481 | 0.1670 | 0.2264 | 0.1704 |  |
|  | 3.8965 | 0.1765 | 0.2336 | 0.1792 |  |
|  | 3.6857 | 0.1807 | 0.2474 | 0.1836 |  |
|  | 3.5058 | 0.1841 | 0.2266 | 0.1862 |  |
|  | 3.3499 | 0.1883 | 0.2478 | 0.1917 |  |
|  | 3.2131 | 0.2035 | 0.2196 | 0.2045 |  |
|  | 3.0918 | 0.2026 | 0.2529 | 0.2049 |  |
|  | 2.9833 | 0.2004 | 0.2346 | 0.2019 |  |
|  | 2.8855 | 0.2099 | 0.2399 | 0.2114 |  |
|  | 2.7967 | 0.2082 | 0.2503 | 0.2101 |  |
|  | 2.7156 | 0.2144 | 0.2216 | 0.2147 |  |
|  | 2.6412 | 0.2662 | 0.2737 | 0.2667 |  |
|  | 2.5725 | 0.3619 | 0.2506 | 0.3583 |  |
|  | **Overall** | 0.1987 | 0.2469 | 0.2012 |  |

# Rxpct factors in resolution bins at cycle 3


|  |  |  |  |  |  |  |  |  |
| --- | --- | --- | --- | --- | --- | --- | --- | --- |
|  | **<d> (Å)**  Rxpct\_Work | Rxpct\_Free | Rxpct\_All | |  |  |  |  |
|  | 16.0655 | 0.3808 | 0.4950 | 0.3845 |  |
|  | 9.2754 | 0.2172 | 0.2740 | 0.2205 |  |
|  | 7.1847 | 0.1933 | 0.2326 | 0.1956 |  |
|  | 6.0722 | 0.1965 | 0.2522 | 0.1989 |  |
|  | 5.3552 | 0.1620 | 0.2766 | 0.1667 |  |
|  | 4.8439 | 0.1515 | 0.2098 | 0.1549 |  |
|  | 4.4558 | 0.1508 | 0.2166 | 0.1542 |  |
|  | 4.1481 | 0.1553 | 0.2239 | 0.1593 |  |
|  | 3.8965 | 0.1653 | 0.2292 | 0.1684 |  |
|  | 3.6857 | 0.1729 | 0.2485 | 0.1763 |  |
|  | 3.5058 | 0.1752 | 0.2176 | 0.1774 |  |
|  | 3.3499 | 0.1785 | 0.2380 | 0.1819 |  |
|  | 3.2131 | 0.1906 | 0.2161 | 0.1921 |  |
|  | 3.0918 | 0.1917 | 0.2359 | 0.1937 |  |
|  | 2.9833 | 0.1926 | 0.2231 | 0.1939 |  |
|  | 2.8855 | 0.2053 | 0.2394 | 0.2071 |  |
|  | 2.7967 | 0.2063 | 0.2419 | 0.2079 |  |
|  | 2.7156 | 0.2108 | 0.2430 | 0.2124 |  |
|  | 2.6412 | 0.2648 | 0.2691 | 0.2651 |  |
|  | 2.5725 | 0.3551 | 0.2849 | 0.3528 |  |
|  | **Overall** | 0.1880 | 0.2423 | 0.1907 |  |
